# *In vivo* dosimetry of thyroid doses from different irradiated sites in children and adolescents: a cross-sectional study

**DOI:** 10.1186/1748-717X-9-40

**Published:** 2014-01-30

**Authors:** Cassiane Cardoso Bonato, Henrique Bregolin Dias, Michele da Silva Alves, Lucas Ost Duarte, Telpo Martins Dias, Maiara Oliveira Dalenogare, Claudio Castelo Branco Viegas, Regina Helena Elnecave

**Affiliations:** 1 Graduate Program in Internal Medicine, Endocrinology, Universidade Federal do Rio Grande do Sul (UFRGS), Porto Alegre, RS, Brazil; 2 Radiation Therapy Center, Hospital de Clínicas de Porto Alegre (HCPA), Porto Alegre, RS, Brazil; 3 Ionizing Radiation Quality Program, Brazilian National Cancer Institute (INCA) José de Alencar Gomes da Silva, Ministério da Saúde, Rio de Janeiro, RJ, Brazil; 4 Endocrinology Service, Hospital das Clínicas de Porto Alegre, Ramiro Barcelos, 2350, 12, 4° andar, Porto Alegre, RS CEP 90035003, Brazil; 5 Universidade Federal do Rio Grande do Sul (UFRGS), Hospital de Clínicas de Porto Alegre (HCPA), Ramiro Barcelos, 2350, Porto Alegre, RS CEP 90035-003, Brazil

**Keywords:** Thyroid gland, Thermoluminescent dosimetry, Radiotherapy, Child, Adolescent

## Abstract

**Background:**

Scattered radiation can be assessed by *in vivo* dosimetry. Thyroid tissue is sensitive to radiation, even at doses <10 cGy. This study compared the scattered dose to the thyroid measured by thermoluminescent dosimeters (TLDs) and the estimated one by treatment planning system (TPS).

**Methods:**

During radiotherapy to sites other than the thyroid of 16 children and adolescents, seventy-two TLD measurements at the thyroid were compared with TPS estimation.

**Results:**

The overall TPS/TLD bias was 1.02 (95% LA 0.05 to 21.09). When bias was stratified by treatment field, the TPS overestimated TLD values at doses <1 cGy and underestimated them at doses >10 cGy. The greatest bias was found in pelvis and abdomen: 15.01 (95% LA 9.16 to 24.61) and 5.12 (95% LA 3.04 to 8.63) respectively. There was good agreement in orbit, head, and spine: bias 1.52 (95% LA 0.48 to 4.79), 0.44 (95% LA 0.11 to 1.82) and 0.83 (0.39 to 1.76) respectively. There was small agreement with broad limits for lung and mediastinum: 1.13 (95% LA 0.03 to 40.90) and 0.39 (95% LA 0.02 to 7.14) respectively.

**Conclusions:**

The scattered dose can be measured with TLDs, and TPS algorithms for outside structures should be improved.

## Background

The effects of ionizing radiation on the thyroid gland have been studied for decades [1,2] and the association between hypothyroidism, hyperthyroidism, thyroid nodules, thyroid cancer and radiation is often reported [3,4]. Nevertheless, the thresholds of absorbed dose, the mechanism of injury, and related risk factors have not been clearly established and necessitate further research. The thyroid is particularly sensitive to radiation and may be directly or indirectly exposed to it during radiation therapy of other organs [5]. Doses as low as 10 cGy are known to be associated with an increased incidence of thyroid nodules and thyroid cancer [6,7].

Children are more sensitive to injury caused by ionizing radiation, due to their greater rate of cell replication and to their longer life expectancy. Furthermore, the distances between body segments in relation to the site of irradiation are smaller in children. Even the most modern radiation therapy will cause incidental exposure of nontarget tissues and organs. Dosimetry studies have shown that radiation scatters to the thyroid gland [8,9].

Knowing the radiation dose that reaches healthy tissues plays a key role in determining clinical effects over time and establishing respective tolerance doses. Older data on damage to normal tissues by radiation therapy were based on retrospective studies or on the clinical experience of radiation therapists [10]. Modern computed tomography (CT)-assisted techniques for planning of three-dimensional conformal radiation therapy enable mathematical estimation of the radiation dose scattered to healthy tissues near the target field during treatment, by means of absorbed radiation dose and volume of the irradiated organ. The Quantitative Analysis of Normal Tissue Effects in the Clinic (QUANTEC) study provided information on dose, volume, and prognosis for different organs, including – more recently – the thyroid gland, while taking into account the development of hypothyroidism [11,12]. A retrospective study of patients with Hodgkin lymphoma showed that thyroid volume percentages in excess of 10, 20, and 30 Gy (V10, V20, and V30) were significantly associated with hypothyroidism at mean doses of 32 Gy. Thyroid V30 was an independent predictor of the risk of hypothyroidism [13].

The dosimetric accuracy of radiation therapy can be measured by means of in phantom or *in vivo* dosimetry [14]. Among the various techniques available for absolute dosimetry, thermoluminescent dosimeters (TLDs) have been widely used for their simplicity, excellent spatial resolution, and ability to integrate the dose absorbed over a certain period of time [15]. TLDs can be used on the body surface or within body cavities. Most studies that use TLDs focus on quantification of the main beam dose, and scattered dose studies are usually performed in phantoms, not *in vivo*[16]. In the present study, we evaluated the agreement between scattered radiation dose to the thyroid as predicted by a treatment planning system (TPS) for three-dimensional conformal radiation therapy and that measured by TLDs placed on the skin overlying the thyroid region in a sample of children treated for a variety of non-thyroid cancers.

## Methods

### Sample

Sixteen patients with a mean age of 6.99 years (range 1.3–17.7 years) received radiation therapy for a variety of non-thyroid cancers in several regions of the body. Treatments were administered with two linear accelerators: a Siemens Mevatron MD (SN 3054, nominal photon energy 6 MV) and a Varian 23EX (SN 3595, nominal energy 6 and 15 MV) at the radiation therapy center of a large tertiary care hospital.

Skin entrance radiation doses at the thyroid region were measured with TLDs. The dosimeters were Harshaw TLD-100 (Thermo RMP) chips (lithium fluoride doped with magnesium and titanium [LiF:MgTi], dimensions: 3×3×0.9 mm^3^). Eleven patients also underwent three-dimensional radiation therapy planning in which the thyroid region was included in the planning CT scan volume, thus enabling estimation of doses scattered to the gland during treatment. Doses measured by the TLDs were then compared with the estimates produced by the TPS mathematical model.

The sample comprised 16 patients, from whom 102 scattered dose measurements were obtained by TLDs and 72 paired dose estimates were calculated by TPS. A total of 72 measurements were used in this comparison, as five patients either did not undergo CT-based treatment planning or did not have the thyroid gland included in their scans.

### In vivo dosimetry

During treatment, TLDs were placed onto the skin overlying the thyroid isthmus and measured the scattered radiation from each treatment field that reached the thyroid gland. As the thyroid is located only a few millimeters from the surface (skin), one may estimate that the entrance dose (by definition, the absorbed dose at depth of maximum ionization) is the dose absorbed by the gland. Therefore, doses measured by TLDs during treatment provide reliable estimates of the radiation dose deposited in the region of the gland. At least two measurements were obtained for each treatment field.

TLDs were provided and analyzed by the Radiation Therapy Quality Program of the Brazilian National Cancer Institute (INCA) in Rio de Janeiro. One pair of TLDs was used for each treatment field, and the mean of two measured values calculated for analysis. The pair of TLDs was placed into a chrome-nickel steel hemispherical build-up cap (radius 15 mm) to accomplish electronic equilibrium and to ensure maximal readings.

Determination of the dose absorbed into TLDs took into account background radiation (BG), the dose linearity correction factor (k_lin_), and the calibration coefficient (CC) of the TLDs employed, as this was a relative dosimetry system—i.e. doses were obtained by comparison between the TLD and a TLD exposed to a known radiation dose under reference conditions. The energy dependence of TLDs could be neglected, since all measurements were obtained at the same energy of dosimeter calibration. The fading effect was also not taken into account, as all dosimeters were irradiated and read together on the same day.

Under reference conditions for TLD irradiation, the absorbed dose used in the experimental array was 40 cGy. TLDs were placed between two 5 cm-thick acrylic slabs (as a phantom to simulate body mass), at center beam, with a distance of 100 cm between the radiation source and the surface, a (10×10) cm^2^ field, a dose rate of 200 MU/min, and a nominal energy of 6 MV.

TLDs were calibrated by adjusting the measurements in thermoluminescent (TL) signal with the doses provided by the linear accelerator, taking into account a dose of 40 cGy (the reference dose, D_ref_), which was the most expected for the majority of patients. To deliver this dose, the accelerator was set to 164 MU.

The linear accelerator was calibrated with a Farmer-type ionization chamber (PTW TN30004, S/N 244) and a PTW Unidos E electrometer (S/N T10010-00055), in accordance with the IAEA TRS-398 protocol.

TLD readouts were performed in a PCL3 (Fimel) reader at the Radiation Therapy Quality Program, INCA, Rio de Janeiro. Annealing was performed with an EDG1800 oven (EDG Equipamentos, Brazil) and a Fanem 315SE drying oven (Fanem, Brazil).

Dose counts are shown in the reader as a TL signal and then converted to an absorbed dose unit (cGy). This value is then multiplied by the calibration coefficient after subtracting the background radiation (BG).

The calibration coefficient (CC) was calculated with Equation 1:

(1) $$\mathrm{CC}=\frac{D_{\mathrm{ref}}}{\mathrm{TL}‒\mathrm{BG}}$$

The uncorrected dose (D_uncorr_) is the uncorrected dose at the measurement TLD, as per Equation 2:

(2) $$D_{\mathrm{uncorr}}=\mathrm{CC}\left(\mathrm{TL}=\mathrm{BG}\right)$$

**Figure 1:**
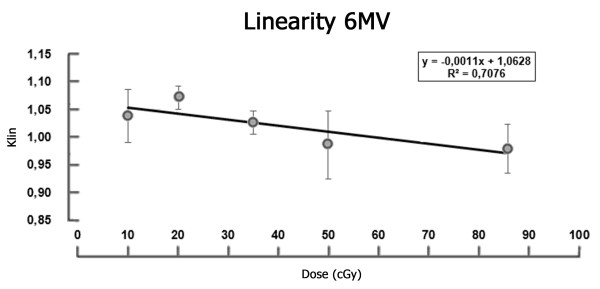
**Linearity coefficient of TLD measurements.** Linearity coefficient of TLD values as a function of absorbed dose in the acrylic phantom.

To correct for nonlinear dose–response, a linearity correction curve was plotted for the radiation beam (Figure 1), using 15 pairs of TLDs in the same experimental array used for irradiation of the calibration TLDs.

Three pairs of TLDs were used for each of the five known doses: 10, 20, 35, 50, and 85 cGy. These doses were chosen in view of the expected doses for the type of treatment used in the study sample. The vertical axis in Figure 1 represents the correction factor for nonlinearity (k_lin_).

The determining points (k_lin_) of the best-fit curve for correction of nonlinearity were calculated using Equation 3 where M is the TLD readout (or TL signal), D is the absorbed dose, and parameters with an index of zero represent reference values or normalization points.

Finally, the patient – or corrected – dose (D_uncorr_) was calculated by the aforementioned factors (Equation 4):

Computerized planning of 3D conformal radiation therapy.

In patients who underwent computerized planning of 3D conformal radiation therapy, a radiologist singled out the thyroid gland in their planning CT scans. The estimated scattered dose to the thyroid during radiation therapy was then calculated with the Eclipse 10.0 TPS (VARIAN Medical Systems, USA). The software package used anisotropic analytical algorithms for dose calculation.

Scattered doses to the thyroid gland and to the skin overlying the thyroid isthmus, where TLDs were placed, were estimated in the TPS. Field and total radiation doses were calculated. Dose-volume histograms were used to determine the minimum, mean, and maximum dose to the gland, and the mean dose was used for statistical analyses.

The distance between the skin overlying the thyroid isthmus to the treatment field hot spot was also measured.

In two patients who received spinal irradiation, part of the main beam dose also contributed to the measured doses.

The procedures carried out with the patients in this study were reviewed and approved by the Research Ethics Committee of the institution where the study was conducted, in accordance with standards set by the committee and in compliance with the 1975 Helsinki Declaration and its 2000 revision. Assent from patients and informed consent from their legal guardians was obtained before the study.

### Statistical analysis

This was a cross-sectional study based on a sample of children and adolescents who underwent treatment at the radiation therapy service of a large tertiary care hospital. The sample comprised 16 patients, from whom 102 scattered dose measurements were obtained by TLDs and 72 paired dose estimates were calculated by TPS. This number is within the range suggested by Altman [17] and Bland, [18] who propose that 50 to 100 observations are required to measure agreement in method comparison studies.

Normally distributed quantitative data were expressed as means and standard deviations. Skewed data were expressed as medians and ranges, and categorical data, as counts and percentages.

To evaluate the difference between TPS estimates of radiation scattered to the thyroid gland (TPS_thyroid_) and to the point of skin overlying the thyroid isthmus (TPS_skin_), where the TLDs were placed, data were log-transformed to reduce asymmetry. Groups were then compared using a linear mixed effects model, which takes into account the fact that repeated observations are available for each patient.

To assess the correlation between TPS_skin_ and TLD, Pearson correlation coefficients were calculated for the log-transformed measurements using a mixed model. Due to the known limitations of the Pearson correlation coefficient for analysis of agreement, we chose to use the Bland–Altman method to compare actual TLD-measured doses of radiation and TPS_skin_ estimates. As TPS_skin_ and TLD values were asymmetrically distributed, we chose to use TPS_skin_/TLD ratios as an alternative to logarithms [19]. Furthermore, only TLD measurements rather than the mean of TPS_skin_ and TLD measurements were plotted onto the x-axis, as recommended when one of the methods under study is considered the gold standard [19]. Results were then plotted on log scale to facilitate visualization of the calculated values. Repeated measures were clustered by patient and analyzed with a mixed effects model, which estimated bias values (mean difference between ratios for each method) and 95% limits of agreement (95% LA).

To assess the impact of prescribed radiation dose and TLD–hot spot distance on TLD measurements, data were log-transformed (to reduce asymmetry), z scores were calculated (to standardize units), and a mixed model was used for analysis.

The significance level was set at α = 0.05. Data were processed and analyzed in the SPSS 18.0, R 2.14.1 and SigmaPlot 11.0 software environments.

## Results

The sample profile is shown in Table 1. Comparison between TPS_thyroid_ and TPS_skin_ values did not reach statistical significance (P = 0.842). Therefore, TPS_skin_ was considered appropriate for comparison with actual TLD-measured doses.

**Table 1:** Sample profile

**Variable**	**n** **=** **16**
Age, years (range)	5.7 (1.3–17.7)
Sex, n (%)	
Male	11 (68.8)
Female	5 (31.3)
Cancer type, n (%)	
Adrenal neuroblastoma	3 (18.8)
Acute lymphoblastic leukemia	2 (12.5)
Retinoblastoma	2 (12.5)
Wilms’ tumor	2 (12.5)
Acute myeloid leukemia	1 (6.3)
Hodgkin’s lymphoma	1 (6.3)
Non-Hodgkin lymphoma	1 (6.3)
Medulloblastoma	1 (6.3)
Mediastinal neuroblastoma	1 (6.3)
Rhabdomyosarcoma	1 (6.3)
CNS tumor	1 (6.3)
Irradiated region, n (%)	
Abdomen	3 (18.8)
Mediastinum	3 (18.8)
Head	2 (12.5)
Head and spine	2 (12.5)
Orbit	2 (12.5)
Lower extremity	1 (6.3)
Pelvis	1 (6.3)
Lung	1 (6.3)
Testicle	1 (6.3)
Thyroid–hot spot distance, cm	14 (4–27)
Total prescribed dose, cGy	3600 (1350–14400)
Scattered dose to the thyroid (TPS), cGy	
Min	92 (9–3780)
Mean	186 (12–4111)
Max	542 (17–4370)
Scattered dose to the thyroid (TLD), cGy	283 (1–6754)
Data are expressed as n (%) or median (range).	

**Figure 2:**
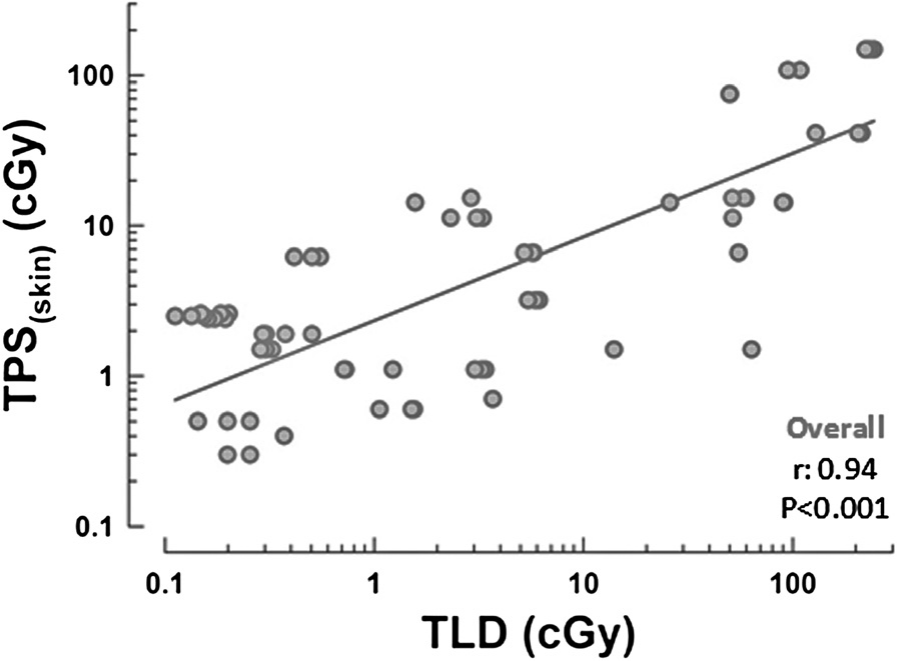
**Scatter plot of correlation between TLD and TPS[skin]-estimated doses.** Scatterplot of relationship between TPS_skin_ and TLD values. r: Pearson correlation coefficient; p: statistical significance.

There was a significant correlation between the TLD-measured dose and the TPS_skin_-estimated dose (r = 0.94, P < 0.001) (Figure 2).

**Figure 3:**
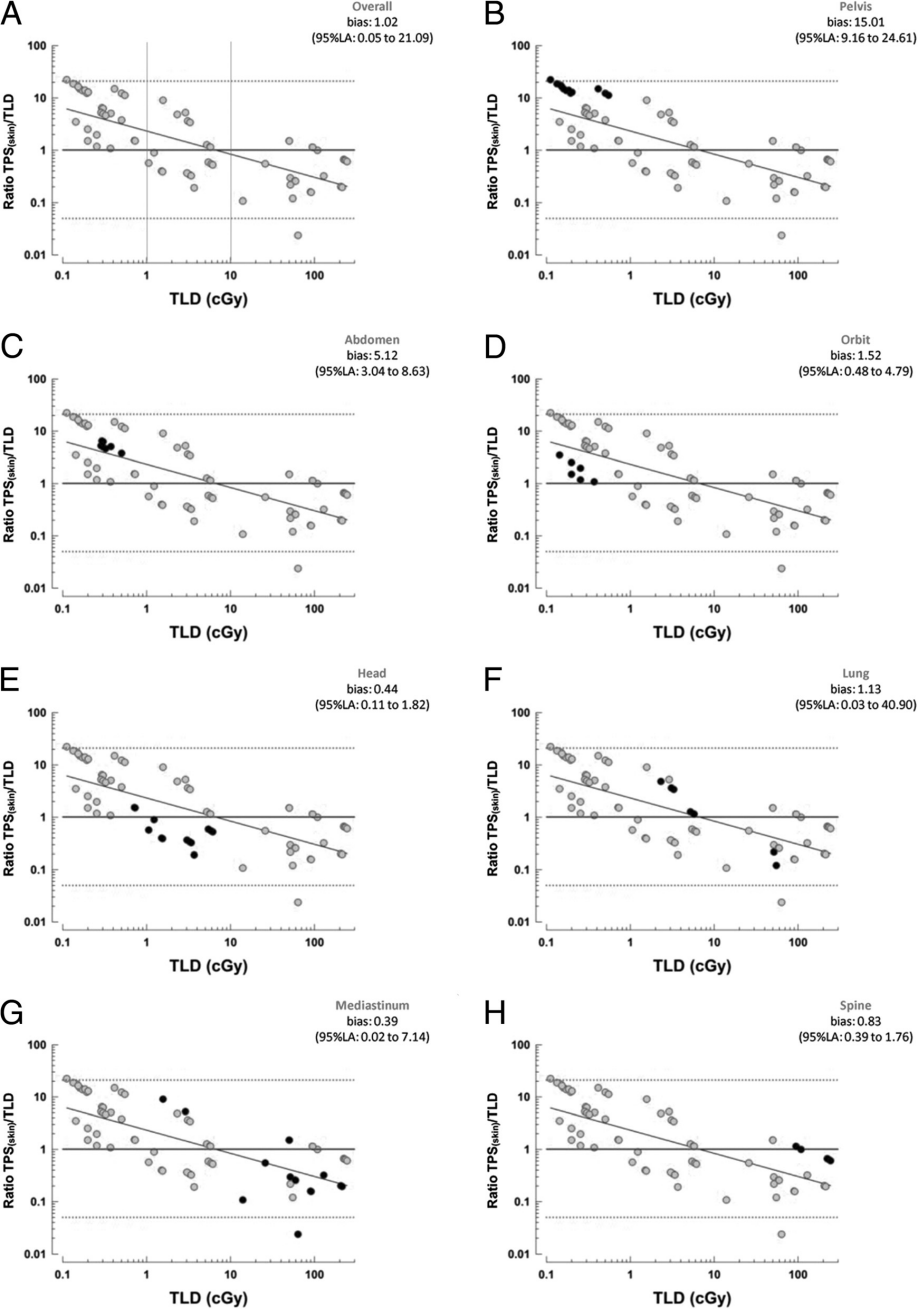
**Bland**–**Altman plots of agreement between TLD and TPS measurements.** Bland–Altman plots of overall agreement between TPSskin and TLD measurements, stratified by specific anatomical site (dark points): **(A)** Overall agreement (bias, 1.02; 95% LA, 0.5 to 21.09); **(B)** Pelvis (bias, 15.01; 95% LA, 9.16 to 24.61); **(C)** Abdomen (bias, 5.12; 95% LA, 3.04 to 8.63); **(D)** Orbit (bias, 1.52; 95% LA, 0.48 to 4.79); **(E)** Head (bias, 0.44; 95% LA, 0.11 to 1.82); **(F)** Lung (bias, 1.13; 95% LA, 0.03 to 40.90); **(G)** Mediastinum (bias, 0.39; 95% LA, 0.2 to 7.14); **(H)** Spine (bias, 0.83; 95% LA, 0.39 to 1.76).

Analysis of Bland-Altman plots for the TPS_skin_/TLD ratio at different levels of radiation showed that the bias of the TPS/TLD ratio behaved differently in relation to TLD-measured doses. Below 1 cGy, the TPS overestimated the actual dose, as measured by TLDs (Figure 3). Between 1 and 10 cGy, the bias ranged around 1, showing agreement between the two methods. Above 10 cGy, the TPS was more likely to underestimate the scattered dose as compared with actual TLD measurements. Although the overall bias was small (bias = 1.02), the 95% limits of agreement were broad (95% LA: 0.05 to 21.09). Analysis by site of irradiation showed different TPS/TLD biases according to the proximity of the thyroid gland to the treatment field.

**Figure 4:**
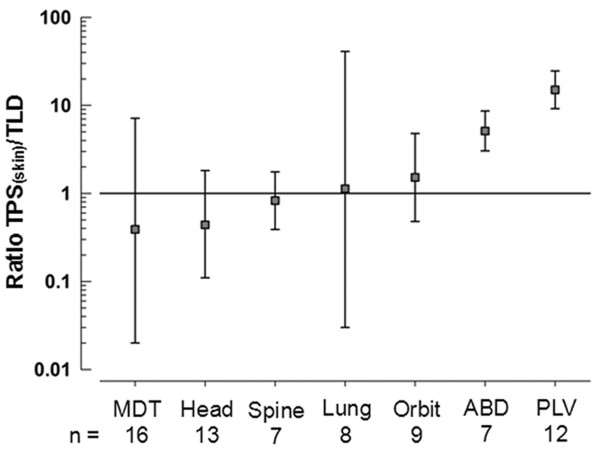
**Limits of agreement for TPS[skin]/TLD ratios by anatomical site.** 95 % limits of agreement for TPS_skin_/TLD ratios, by anatomical site. MDT, mediastinum; ABD, abdomen; PLV, pelvis.

Figures 3 and 4 show dose behavior by site of irradiation. The dose scattered to the thyroid was <0.1 cGy when the pelvis or abdomen were irradiated. The TPS overestimated the scattered dose, and there was little agreement between TPS and TLD measurements—bias of 15.01 (95% LA 9.16 to 24.61) and 5.12 (95% LA 3.04 to 8.63) respectively (Figure 3B and 3C).

The TPS and TLDs provided similar measurements of radiation scattered to the thyroid when the site of irradiation was the orbit, head, or spine, with biases of 1.52 (95% LA 0.48 to 4.79), 0.44 (95% LA 0.11 to 1.82), and 0.83 (95% LA 0.39 to 1.76) respectively (Figure 3D, 3E and 3H).

When the lungs or mediastinum were irradiated, TPS estimates and TLD measurements behaved in a similar fashion, with broad limits of agreement and a bias of 1.13 (95% LA 0.03 to 40.90) and 0.39 (95% LA 0.02 to 7.14) respectively (Figure 3F and 3G). At doses >10 cGy, the TPS underestimated the scattered dose up to tenfold as compared to TLD-measured values.

The TLD detected irradiation of the thyroid region even in patients in whom no TPS had been used and whose treatment site was remote from the thyroid.

The distance between the skin overlying the thyroid isthmus to the treatment field hot spot of radiation therapy had a greater impact on TLD measurements (b=-2.26; P < 0.001) than prescribed dose (b = 1.26; P < 0.001), although the two impacts were independent.

## Discussion

Most dosimetry studies on scattered dose to the thyroid gland are performed in phantom, and are thus retrospective simulations of radiation therapy [8,20]. A previous study conducted *in vivo* dosimetry of the scattered dose to the thyroid using TLDs on adult patients undergoing radiation therapy for breast cancer [21]. In the present study, measurements were obtained in children and adolescents who were receiving radiation for cancers at a variety of sites, with different doses, at different distances from the thyroid. There was significant scatter of radiation to the thyroid gland, even when treatment was targeted at different organs, in different locations, that did not include the thyroid in the treatment field.

TPSs are commonly used to estimate the dose scattered to non-target organs, such as the thyroid gland. We demonstrated that the scattered dose can in fact be measured with TLDs, since we did not find any difference between dose measurements on the skin and TPS-based estimation of doses within the gland. At sites where there was good agreement between TPS and TLD measurements, such as the orbit, head, and spine, TPS estimates are quite reasonable. However, at more remote locations, such as the abdomen and pelvis, the TPS overestimated the scattered dose as compared with actual doses measured by TLDs. The algorithms used for TPS dose estimation are developed for calculation of radiation doses within the treatment field—in fact, only a few centimeters from the field edges—, which also may have influenced bias findings. More elaborate simulations are required to calculate doses to proximal or distal tissues of interest regarding varying volume and density of tissues, solid structures and air cavities. In vivo dosimetry may add information for algorithm calculations, even considering tissue heterogeneity and other biases.

Agreement was low and the limits of agreement broad for the lung and mediastinum because the thyroid was too close to the dosimetric penumbra zone, that is, the edges of the treatment field, where the irradiated dose is 20% to 80% of the prescribed dose at the central axis.

Even when the thyroid was farther from the irradiation field, TLDs measured doses that although small were enough to cause injuring in children and adolescents. Doses as low as 10 cGy are associated with thyroid nodules and cancer [6]. In children, the distance between body segments and the irradiation field is smaller, increasing the scattered radiation to the thyroid gland. Our study showed that the distance from the thyroid to the treatment field hot spot has a greater impact than prescribed dose on TLD measurements, although the two impacts were independent. Considering the heterogeneity among the subjects studied, a larger sample would add to the accuracy of the findings.

## Conclusions

Any exposure of the thyroid gland to radiation is cause for concern, particularly in children. *In vivo* dosimetry plays an important role in the characterization of hazardous exposure with respect to dose escalation. As TPSs are constructed with algorithms that have treatment as their objective, correction of mathematical coefficients for the estimated dose that reaches major structures outside the main treatment field may be enhanced with the knowledge obtained from *in vivo* dosimetry. TLDs and TPS exhibit excellent agreement with radiation doses at the central-axis position, but further studies are required to determine their behavior for different energy peaks outside the central axis. Future studies using *in vivo* TLD dosimetry should be conducted to determine the actual radiation reaching tissues and organs far from the field treated during radiotherapy.

## Abbreviations

TLDs: Thermoluminescent dosimeters; TPS: Treatment planning system; CT: Computed tomography; CC: Calibration coefficient; BG: Background dose.

## Competing interests

This study was sponsored by Conselho Nacional de Desenvolvimento Científico e Tecnológico (CNPq) and Fundo de Incentivo à Pesquisa (FIPE) of Hospital de Clínicas de Porto Alegre (HCPA). The study sponsors had no involvement in the study design, analysis, interpretation of data, writing or submission of this manuscript.

## Authors’ contributions

CCB conceived the study, took part in its design and coordination, performed the statistical analysis and helped draft the manuscript. HBD took part in the design of the study. MSA helped draft the manuscript. LOD took part in its design and coordination and helped draft the manuscript. TMD took part in its design and coordination and helped draft the manuscript. MOD took part in the design of the study. CCBV took part in its design and coordination and helped draft the manuscript. RHE conceived the study, took part in its design and coordination, and helped draft the manuscript. All authors read and approved the final manuscript.
